# Isolation and Quantification of Polyamide Cyclic Oligomers in Kitchen Utensils and Their Migration into Various Food Simulants

**DOI:** 10.1371/journal.pone.0159547

**Published:** 2016-07-25

**Authors:** Yutaka Abe, Motoh Mutsuga, Hiroyuki Ohno, Yoko Kawamura, Hiroshi Akiyama

**Affiliations:** 1 National Institute of Health Sciences, Kamiyoga, Setagaya-ku, Tokyo, Japan; 2 Nagoya City Public Health Research Institute, Hagiyama-cho, Mizuho-ku, Nagoya, Japan; Macau University of Science and Technology, MACAO

## Abstract

Small amounts of cyclic monomers and oligomers are present in polyamide (PA)-based kitchen utensils. In this study, we isolated eight PA-based cyclic monomers and oligomers from kitchen utensils made from PA6 (a polymer of ε-caprolactam) and PA66 (a polymer of 1,6-diaminohexane and adipic acid). Their structures were identified using high-resolution mass spectrometry and ^1^H- and ^13^C-nuclear magnetic resonance spectroscopy, and their residual levels in PA-based kitchen utensils and degree of migration into food simulants were quantified by high-performance liquid chromatography/mass spectrometry using purchased PA6 monomer and isolated PA66 monomers, and isolated PA6 and PA66 oligomers as calibration standards. Their total residual levels among 23 PA-based kitchen utensils made from PA6, PA66, and copolymers of PA6 and PA66 (PA6/66) ranged from 7.8 to 20 mg/g. Using water, 20% ethanol, and olive oil as food simulants, the total migration levels of the PA monomers and oligomers ranged from 0.66 to 100 μg/cm^2^ under most examined conditions. However, the total migration levels of the PA66 monomer and oligomers from PA66 and PA6/66 kitchen utensils into 20% ethanol at 95°C were very high (1,700 and 2,200 μg/cm^2^, respectively) due to swelling by high-temperature ethanol.

## Introduction

**Fig 1:**
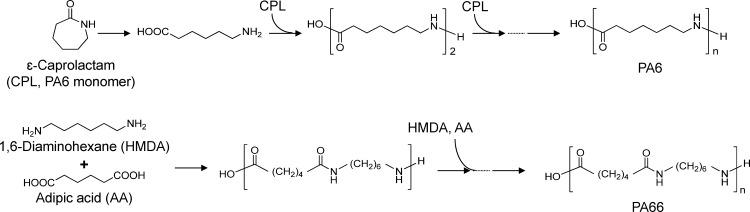
Condensation reactions of PA6 and PA66.

**Fig 2:**
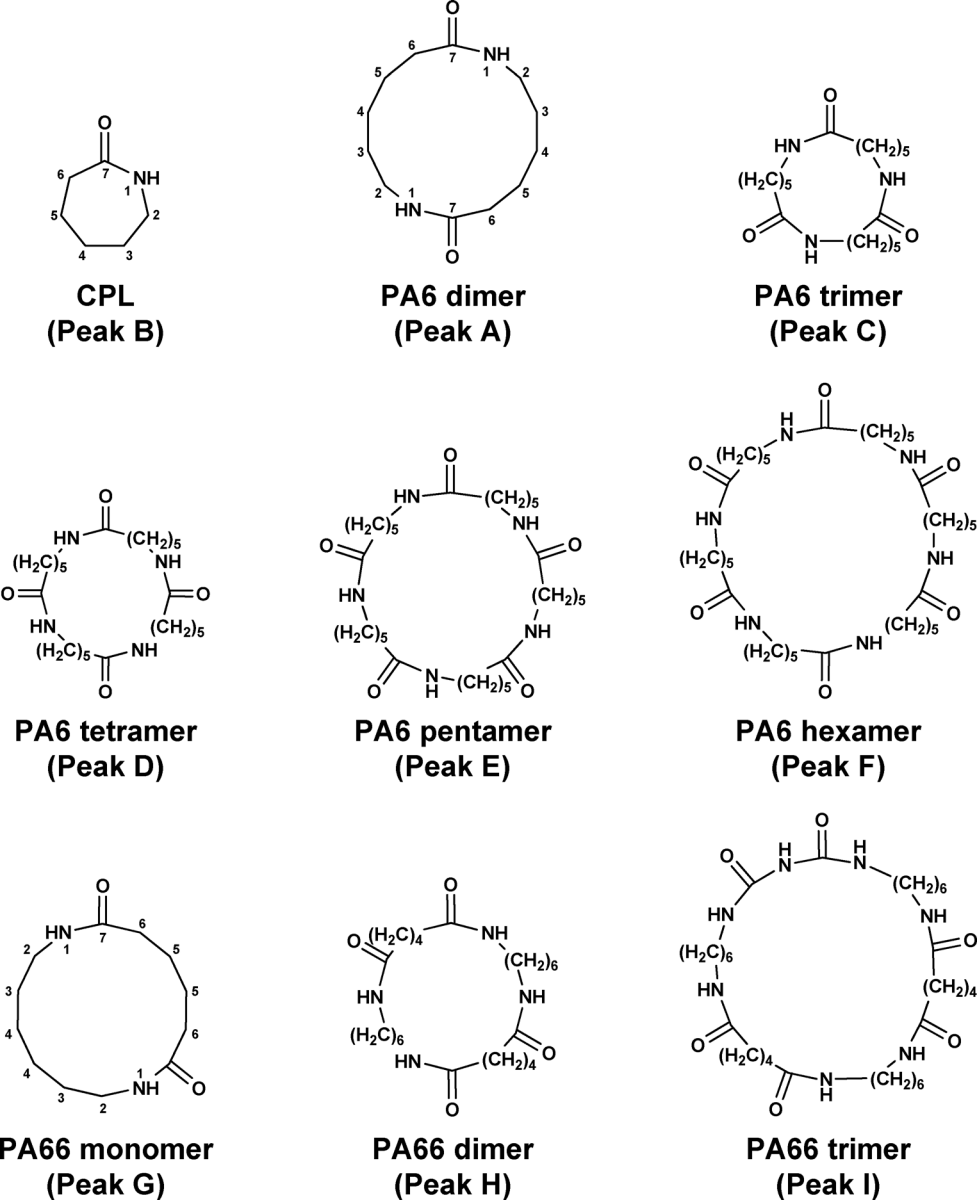
Structures of PA monomers and oligomers. The numbers for CPL, PA6 cyclic dimer, and PA66 cyclic monomer represent the positions shown in Table 1.

Plastic materials are widely used in kitchen utensils such as dishes, cups, spoons, forks, measuring cups, and bottles. Among the various plastics used, polyamide (PA) materials are typically used for turners or ladles due to their high heat and oil resistance. There are many types of PA-based materials, such as PA6, PA66, and PA6/66 (copolymers of PA6 and PA66), all of which are commonly used in kitchen utensils. PA6 is produced by the ring-opening polymerization of ε-caprolactam (CPL), while PA66 is produced by the polycondensation of 1,6-diaminohexane (1,6-hexamethylenediamine, HMDA) and adipic acid (AA) (Fig 1). Small amounts of PA cyclic monomers and oligomers are produced as by-products during these processes and remain in the PA-based materials. Typical structures of PA cyclic monomers and oligomers are shown in Fig 2. For succinctness, we use the term ‘PA66 monomer’ for the cyclic condensation product of HMDA or AA.

**Table 1:** ^1^H- and ^13^C-NMR data of PA cyclic monomers and oligomers.

Monomers and oligomers	Nucleus	Chemical shift data (ppm)						
		1	2	3	4	5	6	7
**PA6 monomer(CPL)**	^1^H	7.60 (s)	3.23 (2H, m)	1.64–1.70^a^ (m)	1.80 (2H, m)	1.64–1.70^a^	2.47	-
						(m)	(2H, t)	
	^13^C	-	44	30.8	31.8	24.5	37.6	182
**PA6 dimer**	^1^H	7.59 (s)	3.27 (4H, t)	1.28 (4H, m)	1.63 (4H, m)	1.48	2.2	-
						(4H, m)	(4H, t)	
	^13^C	-	40	30.1	26.7	26.4	37.1	176.5
**PA6 trimer**	^1^H	7.60 (s)	3.21 (6H, t)	1.34 (6H, m)	1.64 (6H, m)	1.53	2.22	-
						(6H, m)	(6H, t)	
	^13^C	-	40.3	29.6	27	26.3	37	176.4
**PA6 tetramer**	^1^H	7.59 (s)	3.20 (8H, t)	1.33 (8H, m)	1.64	1.52	2.21	-
					(8H, m)	(8H, m)	(8H, t)	
	^13^C	-	40.4	30.1	27.4	26.7	37.2	176.3
**PA6 pentamer**	^1^H	7.60 (s)	3.18 (10H, t)	1.33 (10H, m)	1.63	1.52	2.19	-
					(10H, m)	(10H, m)	(10H, t)	
	^13^C	-	40.5	30.3	27.6	26.8	37.4	176.1
**PA6 hexamer**	^1^H	7.60 (s)	3.20 (12H, t)	1.34 (12H, m)	1.64	1.53	2.21	-
					(12H, m)	(12H, m)	(12H, t)	
	^13^C	-	40.4	30.1	27.4	26.7	37.2	176.3
**PA66 monomer**	^1^H	7.58^a^	3.22 (4H, t)	1.53 (4H, s)	1.32	1.63	2.19	-
					(4H, s)	(4H, s)	(4H, s)	
	^13^C	-	39.9	29.1	27.4	27.3	37.5	176.5
**PA66 dimer**	^1^H	7.59 (s)	3.15 (8H, t)	1.47 (8H, t)	1.31	1.6	2.18	-
					(8H, s)	(8H, s)	(8H, t)	
	^13^C	-	40.4	30.4	27.5	26.7	37.2	176
**PA66 trimer**	^1^H	7.58^a^	3.17 (12H, t)	1.50 (12H, s)	1.34	1.62	2.21	-
					(12H, s)	(12H, s)	(12H, t)	
	^13^C	-	40.5	30.4	27.6	26.7	37.1	175.9

^a^Overlapped with each other.

The data were obtained in CD_3_OD:CDCl_3_ (1:1) solution.

It has been reported that such monomers and oligomers migrate into food and food simulants from food packaging [1–4]. In these reports, PA cyclic monomers and oligomers were analyzed using high-performance liquid chromatography (HPLC) equipped with an ultraviolet (UV) detector at 210 nm [2–6]. Due to a lack of commercially available standards, the quantification of these cyclic oligomers was performed using relative response factors (RRFs) with reference to CPL. Unfortunately, the RRFs reported in previous publications are not consistent. Notably, Heimrich et al. [7] derived cyclic oligomer RRFs (UV 210 nm) relative to CPL using synthesized cyclic oligomers as standards, and their quantification was carried out using an HPLC equipped with a chemiluminescence nitrogen detector (CLND) and UV detector. However, CLND is not commonly available in many laboratories, and the selectivity and sensitivity of a UV detector may be insufficient for the determination of these oligomers.

In this study, we isolated eight PA cyclic monomer and oligomers from PA6- and PA66-based kitchen utensils and identified their structures using high-resolution mass spectroscopy (HRMS) and ^1^H- and ^13^C-nuclear magnetic resonance (NMR) spectroscopy. Furthermore, their residual levels in PA6-, PA66-, and PA6/66-based kitchen utensils were quantified by liquid chromatography/mass spectrometry (LC/MS) using purchased CPL and isolated PA66 monomer and PA6 and PA66 oligomers as calibration standards. In addition, migration tests were performed using various food simulants, e.g., water was used for common foods, 20% ethanol for alcoholic beverages, and olive oil for fat and fatty foods.

## Materials and Methods

### Samples and reagents

Twenty-three kitchen utensils composed of PA (turners, ladles, a sesame grinder, a cake server, and a cake scraper) were purchased at retail stores, such as super markets, one-coin shops, variety store, in Setagaya (Tokyo) and Nagoya (Aichi), Japan. The sample information, such as color, labeling (material types, maximum usage temperature, and producing country), and their material types identified using LC/MS chromatograms of their extracts as mentioned in the ‘Results and Discussion’ section were shown in Table 2. The names of the manufacturing or sales companies of these samples were shown in S1 Table.

**Table 2:** PA kitchen utensil sample types.

Sample	Color	Label			Material*
		Material	Maximum usage temperature (°C)	Producing country	
Turner 1	Black	Nylon 66	180	Japan	66
Turner 2	Gray	Nylon	210	Japan	66
Turner 3	Dark gray	Nylon	180	China	66
Turner 4	Black	Nylon 66	210	Japan	66
Turner 5	Black	Nylon 66	220	Japan	66
Turner 6	Black	Nylon 66, Glass fiber 15%	220	Portugal	66
Turner 7	Black	Nylon	180	Japan	66
Turner 8	Black	Nylon	200	Japan	66
Turner 9	Orange	Nylon	200	China	66
Turner 10	Yellow-green	Nylon	180	China	6/66
Ladle 1	Gray	Nylon	180	China	6
Ladle 2	Dark gray	Nylon	180	China	66
Ladle 3	Black	Nylon 66	210	Japan	66
Ladle 4	Gray	Nylon 66	210	Japan	66
Ladle 5	Black	Nylon	210	China	6/66
Ladle 6	Black	Nylon, Glass fiber 15%	240	China	66
Ladle 7	Black	Nylon 66	200	China	66
Ladle 8	Black	Nylon	200	Japan	66
Ladle 9	Orange	Nylon	180	Japan	66
Ladle 10	Orange	Nylon 66	200	Japan	66
Sesame grinder	White	Nylon	90	Vietnam	6
Cake server	Gray	Nylon	180	China	6/66
Cake scraper	Gray	Nylon	180	China	6/66

*Determined using LC/MS.

ε-Caprolactam (99%) was purchased from Tokyo Chemical Industry Co., Ltd. (Tokyo, Japan). Methanol for HPLC analysis grade (> 99.8%) was obtained from Kanto Chemical Co., Inc. (Tokyo, Japan), and formic acid for HPLC analysis grade (ca. 99%) was obtained from Wako Pure Chemical Industries, Ltd. (Osaka, Japan). Ultrapure water was purified with the Milli-Q Gradient system equipped with a Millipak 0.22 μm filter (Millipore, Billerica, MA, USA).

### LC/MS conditions

The LC/MS was an Acquity Series LC/MS system (Waters, Milford, MA, USA) equipped with an Acquity BEH C18 (100 mm × 2.1 mm, 1.7 μm, Waters). The LC/MS conditions were as follows: column temperature of 40°C; flow rate of 0.25 mL/min; mobile phase of methanol/0.1% formic acid (5:95) (1 min) running with a linear gradient to methanol/0.1% formic acid (95:5) (5 min) and methanol/0.1% formic acid (95:5) (10 min); MS electrospray capillary voltage of 3 kV; drying gas temperature of 400°C; flow rate of 600 L/hour (N_2_), cone gas flow rate of 50 L/hour (N_2_); electrospray ionization (positive) mode, SCAN (*m/z* 50–1000) or single ion recording (SIR) modes; and cone voltage of 30 V (SCAN mode). The SIR mode cone voltage and quantitative ions are shown in Table 3.

**Table 3:** Retention times and MS (SIR mode) conditions for quantification.

Peak	Cyclic monomers and oligomers	Retention time (min)	Quantitative ion (*m/z*)	Cone voltage (V)
Peak A	PA6 dimer	4.2	227.3	30
Peak B	PA6 monomer (CPL)	4.4	113.6	30
Peak C	PA6 trimer	4.9	340.4	30
Peak D	PA6 tetramer	5.3	453.3	30
Peak E	PA6 pentamer	5.5	566.4	40
Peak F	PA6 hexamer	5.7	701.8	40
Peak G	PA66 monomer	4.5	227.3	30
Peak H	PA66 dimer	5.6	453.3	30
Peak I	PA66 trimer	5.9	701.8	40

### Isolation and purification of PA cyclic monomers and oligomers

Three grams of the PA-based samples (PA6: ladle 1; PA66: turner 1) were powdered with liquid nitrogen using a frost shattering machine (JFC-300, Japan Analytical Industry Co., Ltd., Tokyo, Japan). Monomers and oligomers were extracted from the samples with methanol (30 mL) at 40°C overnight and the residue was subsequently removed by filtration with a cotton plug. The filtrates and washings were combined and evaporated using a rotary evaporator (EYERA, Tokyo Rikakikai Co., Ltd., Tokyo, Japan), and the resulting residue was dissolved in 10% methanol. The solution was subjected to chromatography using an OASIS HLB cartridge (6 g, Waters), which was pretreated with methanol and water. Subsequently, each 50 mL of 10, 20, 30, 40, and 50% methanol was passed through the cartridge and collected separately. The eluted and crudely purified monomers and oligomers in each fraction were determined using LC/MS (SCAN mode), and the solvent was subsequently evaporated. Next, using an OASIS HLB cartridge (200 mg, Waters), each 50 mL of 10, 20, 30, 40, and 50% methanol was passed through the cartridge and every 10 mL was collected, and isolated monomers and oligomers in each fraction were determined in the same way. These steps were repeated for each fraction until pure PA cyclic monomers and oligomers were obtained.

CPL and PA6 could not be separated using this cartridge. To separate them, the hydrolysis of CPL and subsequent separation were performed. To hydrolyze the PA6 monomer to 6-aminohexanoic acid (AHA) and separate it from the PA6 dimer, 10 mL of 6 M HCl was added to the residue containing a mixture of PA6 monomer and PA6 dimer (50 mg). The mixture was subsequently heated at 110°C for 6 h and then neutralized with 10% NaOH. This solution was subjected to the OASIS HLB cartridge (200 mg) again, and the PA6 dimer was separated from AHA via elution with 10%–30% methanol.

### Characterization of PA cyclic monomer and oligomers

NMR spectra were recorded on a JEOL Delta 800 spectrometer (800 MHz for ^1^H, 200 MHz for ^13^C) (JEOL Ltd., Tokyo, Japan) using a 1:1 (v/v) mixture of chloroform-*d*/methanol-*d*_4_ as the solvent. To elucidate the structures of the monomer and oligomers, 1D (^1^H and ^13^C) and 2D H-H Correlation Spectroscopy (COSY), ^1^H-Detected Multiple Quantum Coherence (HMQC), and ^1^H-detected Multi-Bond Heteronuclear Multiple Quantum Coherence (HMBC) NMR analyses were performed. Analysis by HRMS was performed by injecting the extracts from ladle 1 and turner 1 into a LC/time-of-flight (TOF)-MS (Aquity LC system, Micromass LCT Premier XE, Waters) system. The LC/TOF-MS conditions were the same as those used for LC/MS.

The purity of each isolated monomer and oligomer was calculated as their peak purities on the total ion chromatograms (TIC) of LC/MS (SCAN mode ranging from *m/z* 50–1,000). The peak purities were calculated by dividing the peak area of each isolated monomer or oligomer by the total peak area on the TIC.

### Quantification of residual levels

For the quantification of the residual levels of PA cyclic monomers and oligomers in kitchen utensils, and to efficiently extract the oligomers from the samples, suitable extraction solvents were selected among water, 50% methanol, methanol, 20% ethanol, ethanol, 2-propanol, and acetonitrile, which are all commonly used extraction solvents. Methanol was found to be the most efficient for the extraction of PA monomers and oligomers. A powdered sample of kitchen utensils (100 mg) using a frost shattering machine was suspended in methanol (5 mL) and heated overnight at 40°C. After filtration using filter paper (No. 5C), the residue was washed with methanol. The filtrates and washings were combined, and the final volume was adjusted to 10 mL with methanol. The test solution was prepared by diluting this solution 20 times with 50% methanol containing 0.1% formic acid. When the concentration of the monomers or oligomers in the test solution exceeded the calibration range, the test solution was diluted 400- or 2,000-fold with the same solution.

### Quantification of migration levels

To quantify the migration levels of PA cyclic monomers and oligomers, migration tests were performed using various food simulants with reference to the Japanese Food Sanitation Law^9)^. In the Law, water and 4% acetic acid were used as the food simulants for common foods based on the pH, 20% ethanol for alcoholic beverages and heptane for fatty foods. Among these simulants, we selected water for common foods, 20% ethanol for alcoholic beverages, and heptane for fatty foods. However, since heptane was not used in the EU as a food simulant, olive oil was used instead.

The migration tests were performed using the total immersing method as follows. The samples were cut into 1 × 5 cm pieces and then placed into screw-capped glass tubes (100 mm i.d. × 300 mm) containing 20 mL of the simulant (water, 20% ethanol, or olive oil), which was preheated to 60°C. The samples were placed in a thermostatically controlled water bath (60°C and 95°C for all simulants) or an autoclave (Autoclave SS-245, Tomy Seiko Co., Ltd., Tokyo, Japan) (121°C for olive oil only) for 30 min. After the migration, the samples were removed from the simulants as soon as possible.

The obtained water migration solutions were added to methanol and formic acid to reach a level of 50% methanol containing 0.1% formic acid, and subsequently subjected to LC/MS. The 20% ethanol migration solutions were subjected to LC/MS without any pretreatment. The olive oil migration solutions were not suitable for LC/MS analysis because of their insolubility in aqueous solutions. Therefore, to analyze these solutions using LC/MS, it was necessary to replace the olive oil with a suitable solvent. This was achieved using the methods reported by Begley et al. [2] and Soto et al. [3]. Briefly, the obtained olive oil solution (5 mL) was added to 50 mL of hexane. It was then transferred into a separatory funnel and 50 mL methanol (saturated with hexane) was added. After shaking the funnel vigorously, the methanol layer was collected and evaporated. The residue was dissolved in 5 mL of 50% methanol containing 0.1% formic acid and was then subjected to LC/MS. When the concentration of the monomers or oligomers in the test solution exceeded the calibration range, the test solution was diluted with the relevant solvent.

### Calibration curve and limit of quantification

To construct calibration curves for the quantification of the residual and migration levels of the PA cyclic monomers and oligomers, standard solutions were prepared as follows. A mixture of the standard stock solution of PA monomers and oligomers was prepared using purchased CPL and isolated PA66 monomer and PA6 and PA66 oligomers with a concentration of 1,000 μg/mL in methanol. The mixtures of standard solutions were prepared with concentrations ranging from 0.001−0.100 μg/mL in 50% methanol containing 0.1% formic acid or 20% ethanol. The prepared mixtures of standard solutions were analyzed using LC/MS, and standard calibration curves were constructed using external standardization. For each PA monomer and oligomer, the limit of quantification (LOQ) was defined as the signal-to-noise ratio of peaks with intensities of 10/1.

## Results and Discussion

### Isolation and characterization of PA cyclic monomer and oligomers for discrimination of polyamide materials

**Fig 3:**
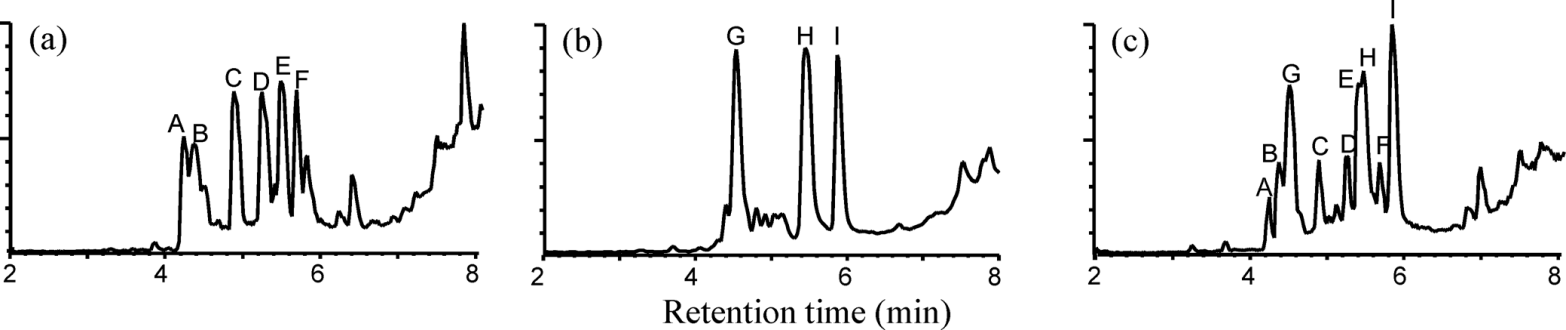
LC/MS total ion chromatograms (SCAN mode) of the extracts. (A) Ladle 1 (PA6), (B) turner 1 (PA66), and (C) cake scraper (PA6/66). LC/MS: liquid chromatography/mass spectrometry.

To identify the polyamide materials, extracts from the samples were subjected to LC/MS (SCAN mode). As shown in Fig 3A–3C, three types of chromatograms were obtained. Among the twenty-three PA-based kitchen utensils, two, seventeen, and four samples exhibited the type of chromatograms shown in Fig 3A, 3B, and 3C, respectively. The compounds corresponding to peaks A, B, C, D, E, and F were detected in the chromatogram shown in Fig 3A, those corresponding to peaks G, H, and I were detected in the chromatogram shown in Fig 3B, and those corresponding to all peaks were detected in the chromatogram shown in Fig 3C.

These compounds were predicted to be PA monomers and oligomers, and by comparing the retention time and mass spectrum of the purchased CPL standard, the compound corresponding to peak B was identified as CPL. To identify the compounds corresponding to peaks A, C, D, E, F, G, H, and I, the compounds were fractionated and purified using a hydrophilic–lipophilic–balanced (HLB) cartridge.

**Fig 4:**
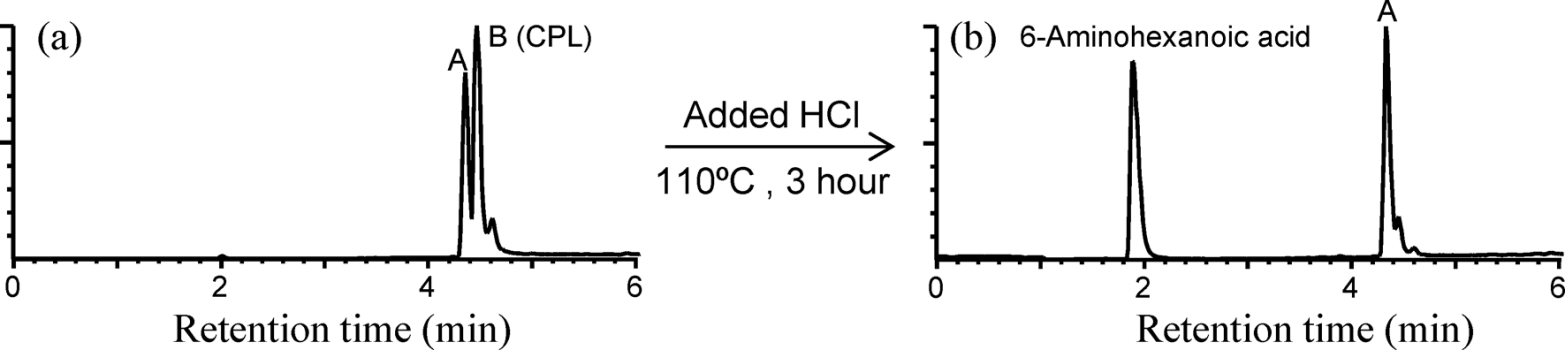
Hydrolysis of CPL to 6-aminohexanoic acid. (A) Before and (B) after hydrolysis.

The compounds corresponding to peaks C (25.3 mg), D (20.3 mg), E (18.7 mg), and F (5.5 mg) were purified from the ladle 1 extracts (Fig 3A), and those corresponding to peaks G (16.4 mg), H (20.2 mg), and I (4.2 mg) were purified from the turner 1 extracts (Fig 3B). As shown in Fig 4A, the compounds corresponding to peak A and CPL were not separable using this cartridge. However, as shown in Fig 4B, the majority of CPL was hydrolyzed to AHA under the described conditions (see the Material and Methods section), and following subsequent purification using the cartridge, the compound corresponding to peak A was successfully obtained (8.0 mg).

**Fig 5:**
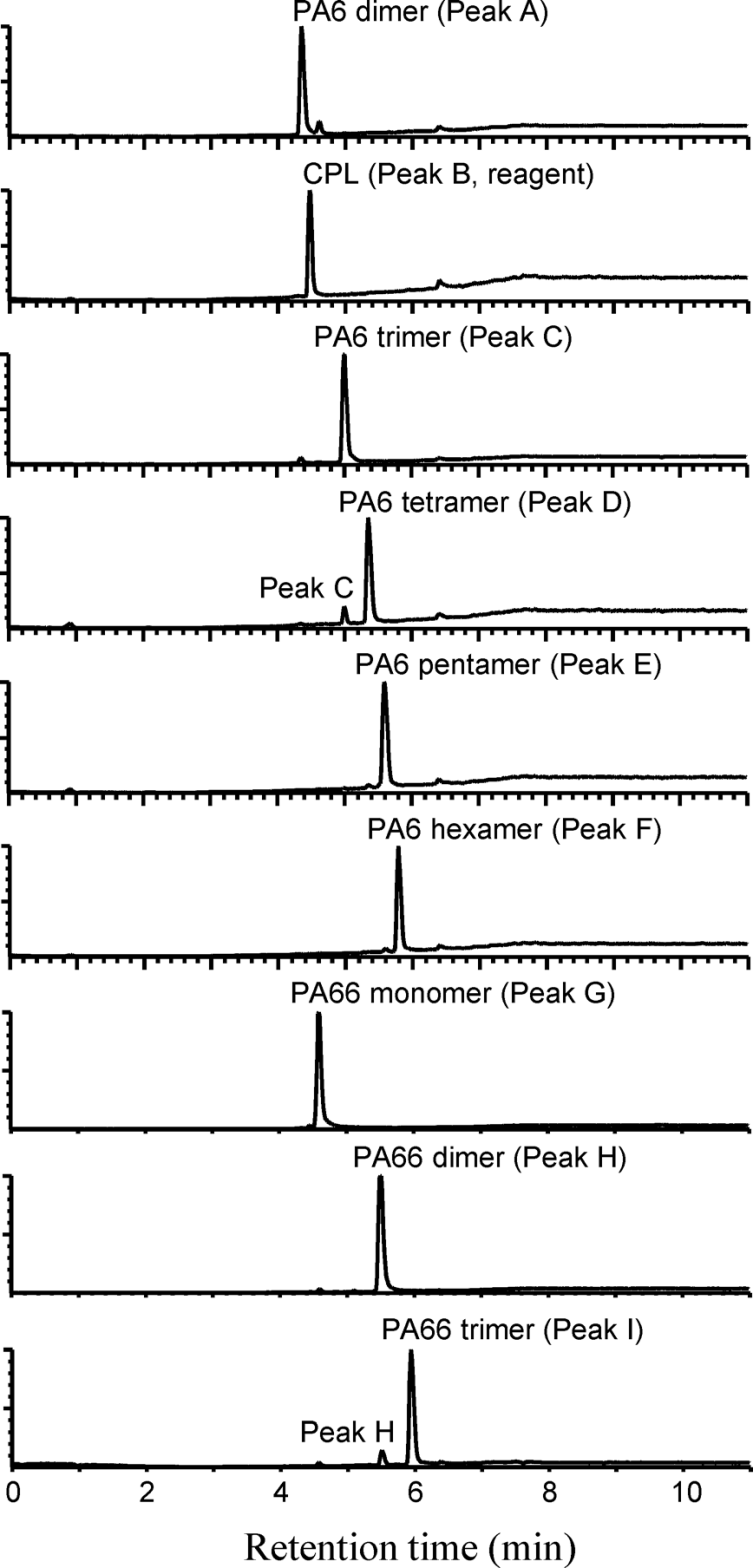
LC/MS total ion chromatograms (SCAN mode) of isolated PA cyclic monomers and oligomers.

To determine the structures of the purified compounds, HRMS and NMR analyses were performed. As shown in Table 4, HRMS revealed that the masses of the compounds corresponding to peaks A, C, D, E, and F were consistent with the calculated theoretical masses of the cyclic dimer, trimer, tetramer, pentamer, and hexamer of CPL, respectively. Furthermore, the masses of the compounds corresponding to peaks G, H, and I were, respectively, consistent with the calculated theoretical masses of the cyclic monomer, dimer, and trimer of HMDA and AA. The 1D and 2D NMR spectra strongly supported the estimated structures (Table 1). These HRMS results showed that the compounds corresponding to peaks A, C, D, E, and F were PA6 cyclic dimer, trimer, tetramer, hexamer, and pentamer, and those corresponding to peaks G, H, and I were PA66 cyclic monomer, dimer, and trimer, respectively (Fig 5). These results also showed that two samples containing only PA6 monomer and oligomers were identified as PA6, seventeen samples containing only PA66 monomer and oligomers were identified as PA66, and four samples containing both PA6 and PA66 monomers and oligomers were identified as PA6/66 on their chromatograms (Fig 3A–3C, Table 2).

**Table 4:** High-resolution mass data of PA6 cyclic oligomers and PA66 cyclic monomer and oligomers.

Peak	Cyclic monomer and oligomers	Formula	Mass (*m/z*)	
			Calculated	Observed
Peak A	PA6 dimer	C_12_H_23_N_2_O_2_	227.1760	227.1779
Peak C	PA6 trimer	C_18_H_34_N_3_O_3_	340.2600	340.2606
Peak D	PA6 tetramer	C_24_H_45_N_4_O_4_	453.3441	453.3427
Peak E	PA6 pentamer	C_30_H_56_N_5_O_5_	566.4281	566.4277
Peak F	PA6 hexamer	C_36_H_67_N_6_O_6_	679.5122	679.5108
Peak G	PA66 monomer	C_12_H_23_N_2_O_2_	227.1760	227.1767
Peak H	PA66 dimer	C_24_H_45_N_4_O_4_	453.3441	453.3438
Peak I	PA66 trimer	C_36_H_67_N_6_O_6_	679.5122	679.5123

As shown in Fig 5, some small peaks corresponding to other oligomers were detected on the TIC of isolated monomers and oligomers, but those corresponding to impurities were not detected. From these results, it was assumed that isolated monomers or oligomers might be slightly mixed in with other oligomers, but not mixed with impurities. These assumptions were confirmed using NMR analysis in that only the signals corresponding to CPL, HMDA, or AA were detected in each NMR spectrum. Thus, the purities of each isolated monomer and oligomer were estimated using the TIC of LC/MS analysis as shown in the Materials and Methods section, and the purities of the PA6 cyclic dimer, trimer, tetramer, pentamer, and hexamer, and PA66 cyclic monomer, dimer, and trimer were estimated to be 84%, 95%, 80%, 93%, 94%, 91%, 84%, and 77%, respectively.

Heimrich et al. [7] reported the identification of four PA linear oligomers and three PA cyclic oligomers (PA6 cyclic dimer, PA66 cyclic monomer, and PA66 cyclic dimer). However, as far as we know, our report is the first that presents the complete identification of the PA6 cyclic trimer, tetramer, and pentamer, and PA66 cyclic trimer. Although we monitored the linear oligomers using LC/MS in the SCAN mode, the linear oligomers could not be detected in this study. This was because the sum of the residual levels of linear oligomers would be less than 2% of those of cyclic oligomers [8].

### Quantification of residual levels of PA cyclic monomers and oligomers in kitchen utensils

The method performance for the quantification of residual levels of PA monomers and oligomers in kitchen utensils was assessed. Good linearity was achieved over the concentration range of 0.001−0.050 μg/mL for the standard solutions of PA monomers and oligomers in 50% methanol containing 0.1% formic acid: correlation coefficients (*R*^*2*^) were > 0.990. The LOQ for the residual levels of PA monomers and oligomers in PA kitchen utensils was estimated to be 0.002 mg/g. The repeatability and reproducibility of the determinations were assessed using the relative standard deviation (RSD) values of intraday (n = 5, RSD_r_) and interday precision (n = 2 × 5 days, RSD_i_) by determining the residual levels of PA monomers and oligomers in the ladle 1 and turner 1 samples. As shown in Table 5, their RSD_r_ values ranged from 4.4% to 6.7%, and RSD_i_ values ranged from 4.8% to 8.6%. These values showed that good linearity, repeatability, and reproducibility were obtained for the quantification of all PA monomers and oligomers.

**Table 5:** Repeatability and reproducibility of the determination method for PA cyclic monomers and oligomers.

Cyclic monomers and oligomers	RSD_r_	RSD_i_
**PA6 monomer (CPL)**	6.1	5.2
**PA6 dimer**	4.7	7.8
**PA6 trimer**	4.4	6.0
**PA6 tetramer**	6.7	5.5
**PA6 pentamer**	6.2	4.8
**PA6 hexamer**	6.3	7.4
**PA66 monomer**	4.7	7.1
**PA66 dimer**	6.2	8.2
**PA66 trimer**	5.7	8.6

RSD_r_ (%): Repeatability (n = 5).

RSD_i_ (%): Reproducibility (n = 2 × 5 day).

As shown in Table 6, the residual levels of PA monomers and oligomers in the 23 kitchen utensil samples were quantified.

**Table 6:** Residual levels of PA cyclic monomers and oligomers in PA kitchen utensils.

Articles	Material type	PA6 cyclic monomer and oligomers							PA66 cyclic monomer and oligomers				Total	PA6:PA66
		Monomer	Dimer	Trimer	Tetramer	Pentamer	Hexamer	Subtotal	Monomer	Dimer	Trimer	Subtotal		
**Ladle 1**	6	4.8	2.8	2.8	4.6	2.8	1.4	19	0.11	0.088	0.063	0.26	19	99:1
**Sesame grinder**	6	3.7	0.8	1.6	5.0	2.8	1.3	15	ND	ND	ND	ND	15	100:0
**Turner 1**	66	0.026	0.013	0.019	0.040	0.011	0.008	0.11	5.4	5.3	3.2	14	14	1:99
**Turner 2**	66	0.003	ND	ND	ND	ND	ND	0.003	4.7	4.5	3.9	13	13	0:100*
**Turner 3**	66	ND	ND	ND	ND	ND	ND	ND	4.4	4.4	3.7	13	13	0:100*
**Turner 4**	66	0.056	0.002	ND	ND	ND	ND	0.057	4.8	4.9	3.9	14	14	0:100*
**Turner 5**	66	0.086	0.006	0.033	0.060	0.016	0.021	0.22	3.0	3.0	2.9	8.9	9.1	2:98
**Turner 6**	66	0.004	ND	ND	ND	ND	ND	0.004	2.8	3.2	3.2	9.1	9.1	0:100*
**Turner 7**	66	0.025	0.005	0.012	0.029	0.010	0.009	0.091	4.7	4.8	4.2	14	14	1:99
**Turner 8**	66	0.062	0.025	0.041	0.088	0.026	0.024	0.27	6.7	6.7	6.2	20	20	1:99
**Turner 9**	66	ND	ND	ND	ND	ND	ND	ND	5.2	5.3	3.4	14	14	0:100*
**Ladle 2**	66	ND	ND	ND	ND	ND	ND	ND	5.3	5.5	4.3	15	15	0:100*
**Ladle 3**	66	0.029	0.005	0.015	0.030	0.011	0.007	0.096	2.7	2.6	2.4	7.7	7.8	1:99
**Ladle 4**	66	0.011	ND	ND	ND	ND	ND	0.011	3.5	3.4	1.9	8.9	8.9	1:99
**Ladle 6**	66	0.12	0.010	0.025	0.050	0.018	0.016	0.24	3.6	4.4	4.2	12	12	2:98
**Ladle 7**	66	0.023	0.005	0.011	0.027	0.009	0.009	0.083	5.2	5.3	4.6	15	15	1:99
**Ladle 8**	66	0.033	0.006	0.013	0.031	0.010	0.009	0.10	2.8	2.8	2.3	7.9	8.0	1:99
**Ladle 9**	66	0.059	ND	ND	ND	ND	ND	0.059	6.0	5.9	4.6	16	17	0:100*
**Ladle 10**	66	0.064	ND	ND	ND	ND	ND	0.064	4.7	4.3	3.4	12	12	1:99
**Ladle 5**	6/66	1.2	0.51	0.60	1.3	0.87	0.58	5.0	1.6	2.1	2.4	6.1	11	45:55
**Turner 10**	6/66	0.63	0.43	0.27	0.80	0.48	0.23	2.8	3.5	3.7	3.2	10	13	22:78
**Cake server**	6/66	1.6	0.32	0.52	1.4	0.82	0.45	5.1	3.7	3.9	3.8	11	17	31:69
**Cake scraper**	6/66	0.74	0.11	0.28	0.58	0.23	0.10	2.1	4.2	4.1	3.7	12	14	15:85

Each value (mg/g) is the mean of two trials.

ND: < 0.002 mg/g

*0:100: PA6 ratios below 0.4.

For the two PA6 samples, the PA6 monomer and oligomers were detected in both samples. Their residual levels ranged from 0.8 to 5.0 mg/g, and their subtotals were 19 and 15 mg/g. The PA66 monomer and oligomers were detected only in ladle 1 with the residual levels ranging from 0.063 to 0.11 mg/g, and their subtotal was 0.26 mg/g.

For the seventeen PA66 samples, the PA66 monomer and oligomers were detected in all samples. Their residual levels ranged from 1.9 to 6.7 mg/g, and their subtotals ranged from 7.7 to 20 mg/g. The PA6 monomer and oligomers were detected in fourteen samples. Their residual levels ranged from 0.003 to 0.12 mg/g and their subtotals ranged from 0.003 to 0.27 mg/g.

For the four PA6/66 samples, the PA6 and PA66 monomers and oligomers were detected in all samples. The residual levels of the PA6 monomer and oligomers ranged from 0.10 to 1.6 mg/g, and their subtotals ranged from 2.1 to 5.1 mg/g. The residual levels of the PA66 monomer and oligomers ranged from 1.6 to 4.2 mg/g, and their subtotals ranged from 6.1 to 12 mg/g. Comparing the subtotals of the residual levels of the PA6 monomer and oligomers with those of the PA66 monomer and oligomers, it is evident that the levels of the PA66 monomer and oligomers were higher.

The total residual levels of PA monomers and oligomers in all samples ranged from 7.8 to 20 mg/g, and there appear to be no significant differences between the material types. Being common to all samples, the residual levels of the PA6 monomer and tetramer appeared to be greater than those of the other oligomers. Those of the PA66 monomer and dimer appeared to be similar while those of the trimer were slightly lower.

### Quantification of migration levels of PA cyclic monomers and oligomers into simulants

Since the quantitative capability for the standard solutions of PA monomers and oligomers in 50% methanol containing 0.1% formic acid was already assessed in the previous section, those in 20% ethanol were assessed in this section. The calibration curves for all of them using 20% ethanol as the standard solution were constructed. All curves had good linearity (0.001−0.050 μg/mL), and the correlation coefficients (> 0.990) and the LOQ for the migration levels in all migration solutions were estimated to be 0.001 μg/mL (0.002 μg/ cm^2^). These results were similar to those of the curve using 50% methanol containing 0.1% formic acid.

The obtained olive oil migration solutions required treatment prior to LC/MS analysis, as described in the Materials and Methods section. To evaluate the performance of the method for determining the migration levels of PA monomers and oligomers in olive oil, recovery tests (n = 3) using 5 mL of olive oil in which were spiked 50 ng of each monomer and oligomer were performed, and the recovery rates and RSD values were assessed. As shown in Table 7, the recovery rates of the PA6 monomer and oligomers ranged from 73% to 105% and those of the PA66 monomer and oligomers ranged from 81% to 95%. The RSD values ranged from 1.1 to 10.2%. These are considered acceptable recovery rates and RSD values for all PA monomers and oligomers, suggesting that the proposed method was reliable for their quantitative determination in olive oil.

**Table 7:** Recovery rates of PA cyclic monomers and oligomers from olive oil.

Cyclic monomers and oligomers	Recovery rate (%)
**PA6 monomer (CPL)**	98 ± 3 (3.3)
**PA6 dimer**	101 ± 2 (2.1)
**PA6 trimer**	105 ± 6 (5.5)
**PA6 tetramer**	103 ± 3 (2.6)
**PA6 pentamer**	87 ± 9 (9.8)
**PA6 hexamer**	73 ± 8 (10.2)
**PA66 monomer**	92 ± 1 (1.1)
**PA66 dimer**	95 ± 7 (7.1)
**PA66 trimer**	81 ± 6 (7.9)

Each value is given as the mean ± SD (RSD%) of three trials.

Each oligomer (50 ng) was spiked in 5 mL of olive oil.

For kitchen utensils that are generally used at room temperature, such as sesame grinders and cake servers, migration tests were performed at 60°C for 30 min (reference to the official Japanese method conditions [9]) using water, 20% ethanol, and olive oil. As shown in Table 8, the results suggested that the PA monomers and oligomers more readily migrated into 20% ethanol than into water but migrated little into olive oil. The migration levels of PA monomers and oligomers decreased with increasing molecular weight. Specifically, there was a strongly negative linear correlation between the migration level and the molecular weight of the PA66 monomer and oligomers into water and 20% ethanol (*R*^*2*^ = 0.987 and 0.990, respectively).

**Table 8:** Migration levels of PA cyclic monomers and oligomers from the sesame grinder (PA6) and cake server (PA6/66) into three simulants at 60°C.

PA cyclic monomers and oligomers	Sesame grinder (PA6)				Cake server (PA6/66)			
	Water	20% Ethanol	Olive oil	Residual level	Water	20% Ethanol	Olive oil	Residual level
**PA6 monomer**	20	21	0.60	3.7	2.5	3.3	0.028	1.6
**PA6 dimer**	2.5	7.7	0.067	0.8	0.49	0.90	0.003	0.32
**PA6 trimer**	3.6	9.8	0.034	1.6	0.54	1.3	0.003	0.52
**PA6 tetramer**	6.2	20	0.008	5.0	0.81	2.1	ND	1.4
**PA6 pentamer**	1.5	6.1	ND	2.8	0.069	0.69	ND	0.82
**PA6 hexamer**	0.15	1.5	ND	1.3	0.025	0.11	ND	0.45
**Subtotal**	34	66	0.71	15	4.5	8.4	0.033	5.1
**PA66 monomer**	ND	ND	ND	ND	9.7	24	0.083	3.7
**PA66 dimer**	ND	ND	ND	ND	4.2	11	0.004	3.9
**PA66 trimer**	ND	ND	ND	ND	0.33	2.3	ND	3.8
**Subtotal**	ND	ND	ND	ND	14	37	0.087	12
**Total**	34	66	0.71	15	19	45	0.12	17

Each value (migration level: μg/cm^2^; residual level: mg/g) is mean of two trials.

ND (migration level): < 0.002 μg/cm^2^; ND (residual level): < 0.002 mg/g.

The migration levels of the PA6 tetramer from the sesame grinder and of the PA66 monomer from the cake server into 20% ethanol were similar to that of CPL from the sesame grinder. The migration limit of CPL to 20% ethanol is regulated in Japan [i.e., migration limit of 15 μg/mL (30 μg/cm^2^)] [9] and in the EU [by regulation 10/2011/EU; specific migration limit of 15 mg/kg)] [10]. However, there are no regulations regarding the migration levels of the PA6 tetramer and PA66 monomer. In Japan, such migration levels are only regulated by a limitation of the total residue upon evaporation [30 μg/mL (60 μg/cm^2^)], and in the EU by the overall migration limit (10 mg/dm^2^) of all migrated substances. The total migration level from the sesame grinder into 20% ethanol is 66 μg/cm^2^, which appears to exceed the Japanese regulation limit. However, the test for the level of residue upon evaporation measures the amount of the residual substance that is not volatilized from the test solution after the solution is evaporated to dryness on a water bath. Under these conditions, some PA monomers and oligomers would be volatilized, so the total level of residue upon evaporation would not exceed the regulatory limit.

For the ladles, which are generally used at temperatures ranging from room temperature to 100°C, the migration tests were performed using a water bath heated at 95°C for 30 min using water, 20% ethanol, and olive oil. Typical results of the migration tests for the three selected samples made from PA6, PA66, and PA6/66 are shown in Table 9. Similar to the migration test performed at 60°C, the PA6 monomers and oligomers more readily migrated into 20% ethanol than into water but migrated little into olive oil.

**Table 9:** Migration levels of PA cyclic monomers and oligomers from ladles 1 (PA6), 8 (PA66), and 5 (PA6/66) into three simulants at 95°C.

PA cyclic monomers and oligomers	Ladle 1 (PA6)				Ladle 8 (PA66)				Ladle 5 (PA6/66)			
	Water	20% Ethanol	Olive oil	Residual level	Water	20% Ethanol	Olive oil	Residual level	Water	20% Ethanol	Olive oil	Residual level
**PA6 monomer**	18	23	0.67	4.8	0.44	0.47	0.071	0.033	18	20	0.44	1.2
**PA6 dimer**	0.85	2.3	0.042	2.8	0.026	0.067	0.023	0.006	1.5	3.2	0.044	0.51
**PA6 trimer**	1.2	3.0	0.025	2.8	0.045	0.12	0.002	0.013	2.3	5.2	0.046	0.60
**PA6 tetramer**	3.4	10	0.017	4.6	0.092	0.30	ND	0.031	3.8	9.7	0.028	1.3
**PA6 pentamer**	3.0	11	ND	2.8	0.007	0.019	ND	0.010	0.16	3.9	ND	0.87
**PA6 hexamer**	1.3	7.4	ND	1.4	0.010	0.036	ND	0.009	0.20	2.1	ND	0.58
**Subtotal**	28	56	0.75	19	0.62	1.0	0.096	0.10	26	44	0.56	5.0
**PA66 monomer**	0.034	ND	0.003	0.11	39	860	0.54	2.8	46	1000	1.6	1.6
**PA66 dimer**	0.031	ND	ND	0.088	20	560	0.018	2.8	26	700	0.15	2.1
**PA66 trimer**	0.008	ND	ND	0.063	5.1	260	ND	2.3	7.2	480	ND	2.4
**Subtotal**	0.073	ND	0.003	0.26	64	1700	0.56	7.9	78	2200	1.7	6.1
**Total**	28	56	0.75	19.3	65	1700	0.66	8.0	104	2200	2.3	11.1

Each value (migration level: μg/cm2; residual level: mg/g) is mean of two trials.

ND (migration level): < 0.002 μg/cm^2^; ND (residual level): < 0.002 mg/g.

On the other hand, the PA66 monomer and oligomers, surprisingly, migrated from ladle 8 (PA66) and ladle 5 (PA6/66) into 20% ethanol at levels ranging from 260 to 1,000 μg/cm^2^. These high levels of migration were probably due to the hydrolysis of the amide linkage of PA66 caused by the swelling ethanol and water.

For turners, which are generally used at temperatures over 100°C, the migration tests were performed at 121°C for 30 min using olive oil. Typical results for the migration tests of three selected samples made from PA66 and PA6/66 are shown in Table 10. For the PA6 monomer and oligomers, the migration levels were roughly correlated with the residual levels. For the PA66 monomer and oligomers, although the migration levels were also correlated with the residual levels, the migration levels drastically decreased with increasing degree of polymerization.

**Table 10:** Migration levels of PA cyclic monomers and oligomers from turners 1 and 2 (PA66), and 10 (PA6/66) into olive oil at 121°C.

PA cyclic monomers and oligomers	Turner 1 (PA66)		Turner 2 (PA66)		Turner 10 (PA6/66)	
	Migration level	Residual level	Migration level	Residual level	Migration level	Residual level
**PA6 monomer**	0.080	0.026	0.070	0.003	0.73	0.63
**PA6 dimer**	0.012	0.013	ND	ND	0.51	0.43
**PA6 trimer**	0.013	0.019	ND	ND	0.17	0.27
**PA6 tetramer**	0.010	0.040	ND	ND	0.16	0.80
**PA6 pentamer**	ND	0.011	ND	ND	0.006	0.48
**PA6 hexamer**	ND	0.008	ND	ND	ND	0.23
**Subtotal**	0.12	0.12	0.070	0.003	1.6	2.8
**PA66 monomer**	6.2	5.4	6.2	4.7	7.3	3.5
**PA66 dimer**	0.97	5.3	0.87	4.5	0.98	3.7
**PA66 trimer**	0.007	3.2	0.007	3.9	0.006	3.2
**Subtotal**	7.2	14	7.1	13	8.3	10
**Total**	7.3	14	7.2	13	9.9	13

Each value (migration level: μg/cm^2^; residual level: mg/g) is mean of two trials.

ND (migration level): < 0.002 μg/cm^2^; ND (residual level): < 0.002 mg/g.

## Conclusions

Five PA6 cyclic oligomers and three PA66 cyclic monomer and oligomers were successfully identified in kitchen utensils composed of PA6 and PA66, and their structures were characterized using HRMS and NMR analyses. Furthermore, the residual and migration levels of PA monomers and oligomers in kitchen utensils were quantified using a purchased PA6 monomer and isolated PA monomer and oligomers.

The total residual levels of PA monomers and oligomers in PA materials ranged from 7.8 to 20 μg/cm^2^, and no significant differences were observed among the total residual levels in PA6, PA66, and PA6/66 materials.

The migration tests suggested that these monomers and oligomers readily migrated into alcoholic beverages possibly due to their features of easy swelling, but hardly migrated into fatty foods possibly due to their low solubility and swelling in the oil. Furthermore, the migrant substances were primarily cyclic monomers and low molecular weight cyclic oligomers. In addition, the PA66-based materials might be easily swelled and degraded, potentially leading to a large amount of PA66 cyclic monomer and oligomers migrating on contact with alcohols at high temperature.

## Supporting Information

S1 Table Supporting information for kitchen utensil samples. (DOCX)
