# Supplementary material for: Isolation and Quantification of Polyamide Cyclic Oligomers in Kitchen Utensils and Their Migration into Various Food Simulants
Source: PLoS One. 2016 Jul 25;11(7):e0159547. doi: 10.1371/journal.pone.0159547 (PMC4959713; doi:10.1371/journal.pone.0159547)
Supplement: S1 Table — (DOCX) [file pone.0159547.s001.docx]

**Supporting information captions**

| **S1 Table. Supporting information for kitchen utensil samples.** | |
| --- | --- |
| **Sample** | **Manufacturing or sales company** |
|  |  |
| Turner 1 | Suncraft Co., Ltd. |
| Turner 2 | DAISO Industries Co., Ltd. |
| Turner 3 | Alpha Corporation Inc. |
| Turner 4 | Echo Kinzoku Co., Ltd. |
| Turner 5 | Echo Kinzoku Co., Ltd. |
| Turner 6 | Group SEB |
| Turner 7 | Pearl Metal Co., Ltd. |
| Turner 8 | Akebono Industry Co., Ltd. |
| Turner 9 | Fujisho Inc. |
| Turner 10 | Unknown |
| Ladle 1 | DAISO Industries Co., Ltd. |
| Ladle 2 | Alpha Corporation Inc. |
| Ladle 3 | Unknown |
| Ladle 4 | Unknown |
| Ladle 5 | DAISO Industries Co., Ltd. |
| Ladle 6 | Ryohin Keikaku Co., Ltd. |
| Ladle 7 | LEBEN Co., Ltd. |
| Ladle 8 | Pearl Metal Co., Ltd. |
| Ladle 9 | Pearl Metal Co., Ltd. |
| Ladle 10 | marna inc. |
| Sesame grinder | Iwasaki Industry Inc. |
| Cake server | DAISO Industries Co., Ltd. |
| Cake scraper | DAISO Industries Co., Ltd. |
